# Lake volume variation in the endorheic basin of the Tibetan Plateau from 1989 to 2019

**DOI:** 10.1038/s41597-022-01711-w

**Published:** 2022-10-08

**Authors:** Junxiao Wang, Liuming Wang, Mengyao Li, Liping Zhu, Xingong Li

**Affiliations:** 1 School of Public Administration, University of Finance & Economics, Nanjing, 210023 China; 2 School of Geography and Ocean Science, Nanjing University, Nanjing, 210023 China; 3 Key Laboratory of Tibetan Environment Changes and Land Surface Processes (TEL), Institute of Tibetan Plateau Research (ITP), Chinese Academy of Sciences, Beijing, 100101 China; 4 Department of Geography & Atmospheric Science, University of Kansas, Lawrence, 66045 United States of America

**Keywords:** Hydrology, Geography

## Abstract

Lake storage change serves as a unique indicator of natural climate change on the Tibetan Plateau (TP). However, comprehensive lake storage data, especially for lakes smaller than 10 km^2^, are still lacking in the region. In this dataset, we completed a census of annual relative lake volume (RLV) for 976 lakes, which are larger than 1 km^2^, on the endorheic basin of the Tibetan Plateau (EBTP) during 1989–2019 using Landsat imagery and digital terrain models. Our method first identifies individual lakes, determines their analysis extents and calculates annual lake area from Landsat imagery. It then derives lake area-elevation relationship, estimates lake surface elevation, and calculates RLV. Validation and comparison with several existing datasets indicate our data are more reliable and comprehensive. Our study complements existing lake datasets by providing a complete and long-term lake water volume change data for the region.

## Abstract (summary)

**Table:** 

Measurement(s)	Relative lake volume
Technology Type(s)	Remote sensing image and DEM data
Sample Characteristic - Environment	endorheic lake
Sample Characteristic - Location	Tibetan Plateau

## Background & Summary

Alpine lakes in arid and semi-arid endorheic watersheds are susceptible to climate change^1,2^. One of the world’s largest alpine lake groups are found on the Tibetan Plateau (TP)^3^, which is often referred to as “the Third Pole of the Earth”^4^ or the “roof and the world”, provides vital water resources for more than a billion population in Asia, and is undergoing rapid climate change^5^.

In the past 50 years, the TP has undergone a much faster warming trend (~0.447 °C per decade) than the global average (0.15–0.20 °C per decade)^6,7^, which posed inevitable impacts on its alpine lakes^8,9^. With little human disturbance in the region, lake water volume change serves as an important indicator that reflects the regional hydrologic system’s responses to climate change^10,11^. Lake area on the TP has been increasing, which is the opposite of the changes in other regions of China^12^, Asia’s plateaus^13^, and other regions or drainage basins across the globe^14^. Furthermore, alpine lakes on the endorheic basins of the Tibetan Plateau (EBTP) have a unique role as they serve as nodes linking the atmospheric, cryospheric, and biospheric components of the hydrological cycle. To understand climate change impacts on the hydrological cycle in the region, it is essential to monitor the volume change of these alpine lakes^15^.

Due to the harsh environment and few *in situ* observation, satellite remote sensing has become an indispensable tool for studying the dynamics of alpine lakes on the TP^16–18^. Satellite imagery has been used for long-term and large-scale monitoring of alpine lakes^1,3,8,16,19–21^ and lake water volume changes on the TP have been examined using Landsat data^12,13,15^. Table 1 summarizes recent studies on lake water volume changes in the region. In the two most recent studies, Li, *et al*.^19^ examined water level and storage changes of 52 lakes with an area larger than 150 km^2^ on the TP using altimetry and optical remote sensing images during 2000–2017. Yao, *et al*.^1^ estimated the relative lake water volume of 871 lakes from 2002–2015 on the Changtang Plateau (CP) in the north-western TP using optical imagery and digital elevation models. Those existing studies, however, are limited to a few large lakes (e.g. lakes larger than 10 km^2^ in Zhang, *et al*.^20^ and lakes larger than 100 km^2^ in Li, *et al*.^19^, specific years (every 5 or 10 years), or a short time span of less than 15 years. There are about 1200 lakes with an area larger than 1 km^2^ in the region^13,22^ and the Landsat earth observation satellite has data archive of more than 40 years^23^. It is necessary to have a long-term census of lake water volume change to study the impacts of climate change on the hydrological cycle in the region. Nevertheless, existing lake studies have neither made full use of available earth observation data, nor have they covered more than 75% of the lakes in the region.

**Table 1:** Recent lake studies and datasets on the TP.

Study	No. of lakes	Temporal resolution	Timespan
Zhang, *et al*.^20^	60–70	One record in the 1970s and annual for 1989–2015	1972–2015
Yang, *et al*.^11^	114	1976, 1990, 2000, 2005 and 2013	1976–2013
Yang, *et al*.^3^	874	Monthly	2009–2014
Yao, *et al*.^1^	871	Annual	2002–2015
Li, *et al*.^19^	52	Monthly	2000–2017
Ke, *et al*.^52^	823	2000 and 2014~14 years	2000 and 2014
Luo, *et al*.^53^	256	Annual	2003–2020
**This study**	976	Annual	1989–2019

In this research, using the Google Earth Engine (GEE) geospatial analysis platform, we analysed 30 years (1989–2019) of Landsat imagery to obtain annual lake area time series for 976 lakes with a maximum area larger than 1 km^2^ on the EBTP. We further estimated annual volume change for the lakes based on the relationship between lake area and surface elevation using digital terrain model data. This study provides a lake water volume dataset which covers so far the largest number of lakes and the longest time span on the EBTP.

## Methods

### Study area and data

**Fig. 1:**
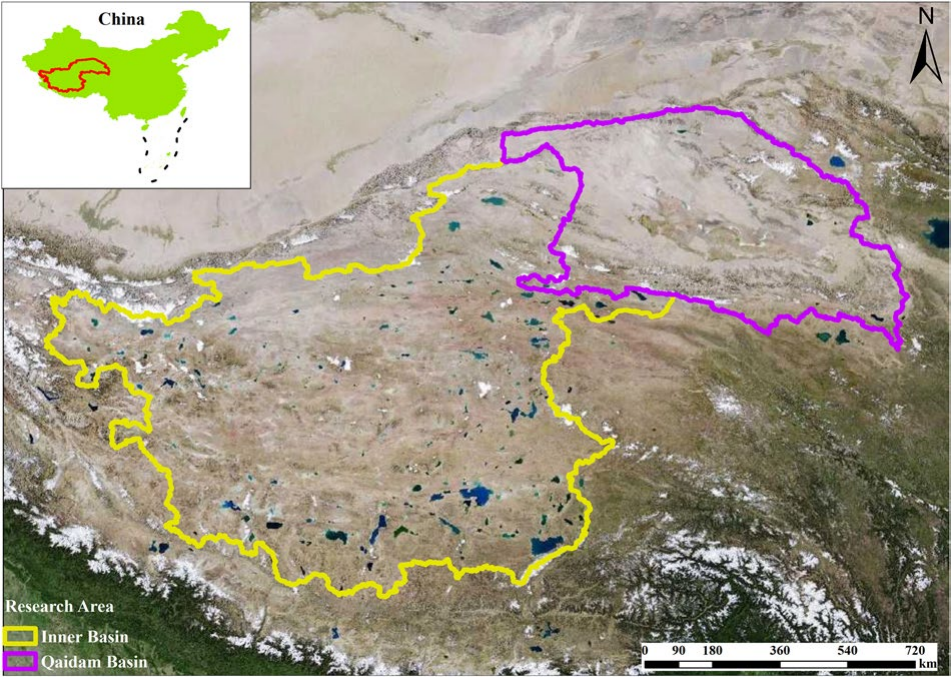
Study area and two sub-regions (the Inner and Qaidam Basins). Background remote sensing image is from http://t0.tianditu.gov.cn/img_c/wmts.

The EBTP (78.646E–99.379E, 29.829N–39.419 N), which has a total area of 1.42 × 10^6^ km^2^, can be generally divided into two sub-basins: the Inner and Qaidam Basins (IB and QB) (Fig. 1). 976 lakes with a maximum area larger than 1 km^2^ in 1989–2019 are selected in this study, which have a total area of 30912.03 km^2^ in 2019.

The data used in this research include Landsat imagery^24–26^, Joint Research Centre Global Surface Water (JRC-GSW) data^27^, Shuttle Radar Topography Mission (SRTM) digital elevation model^28^ (DEM), Advanced Land Observing Satellite (ALOS) digital surface model^29^ (DSM), and several public lake storage data. Both ALOS and SRTM have a spatial resolution of 30 m and cover the study area.

The JRC-GSW dataset contains the distribution of surface water from 1984 to 2020 and provides statistics on the extent and change of those water surfaces^27^. These data were generated using 4,453,989 scenes from Landsat 5, 7, and 8 acquired between 16 March 1984 and 31 December 2020. Each pixel was individually classified into water/non-water using an expert system and the results were collated into a monthly history for the entire time period and two epochs (1984–1999, 2000–2020) for change detection. The product consists of one image containing 7 bands. Areas where water has never been detected are masked. Detailed information for each band is shown in Table 2.

**Table 2:** Bands in Joint Research Centre Global Surface Water data.

Name	Description	Min	Max	Units
occurrence	The frequency with which water was present.	0	100	%
change_abs	Absolute change in occurrence between two epochs: 1984–1999 vs 2000–2019.	−100	100	%
change_norm	Normalized change in occurrence. (epoch1-epoch2)/(epoch1 + epoch2) * 100	−100	100	%
seasonality	Number of months water is present.	0	12	
recurrence	The frequency with which water returns from year to year.	0	100	%
transition	Categorical classification of change between first and last year.	/	/	/
max_extent	Binary image containing 1 anywhere water has ever been detected.	/	/	/

Imagery from Landsat-5 TM (1984–2012), Landsat-7 ETM + (1999−), and Landsat-8 OLI (2013−) were used to extract lake and calculate annual lake area. The JRC-GSW data provide monthly surface water extent from 1984 to 2019 and statistics on the extent and surface water change, which were generated using over 3 million scenes from Landsat 5, 7, and 8^27^. The dataset is used to identify individual lakes and to determine their analysis extents in this study. SRTM and ALOS digital terrain models (DTM) (referring to either DEM or DSM) are used to remove rivers from the water extents derived from JRC-GSW data and to establish the relationship between lake area and water surface elevation.

For validation purpose, we compared our results with a widely used lake surface elevation/storage data from the Laboratoire d’Etudes en Géophysique et Océanographie Spatiales (LEGOS) Hydroweb^30^ and two most recent lake water volume data from Li, *et al*.^19^ and Yao, *et al*.^1^. Only the overlapping lakes from these datasets are used in the comparison.

### Calculating procedure

**Fig. 2:**
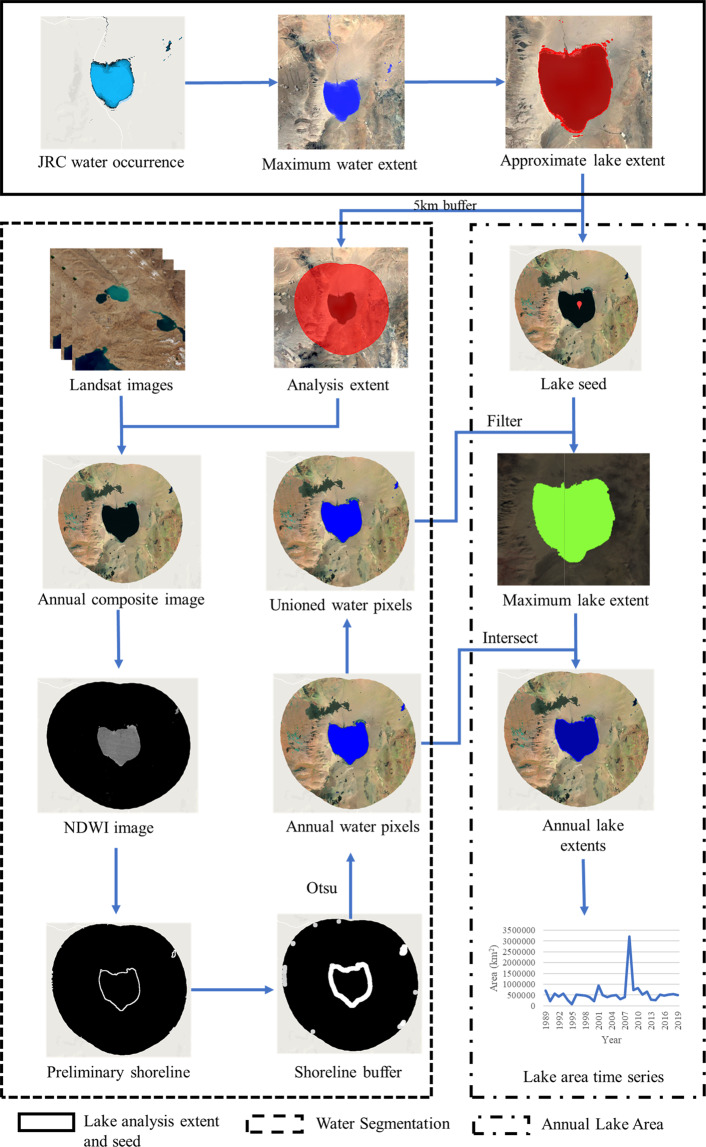
Workflow for calculating annual lake area from Landsat imagery. Background remote sensing image is from http://t0.tianditu.gov.cn/img_c/wmts.

In this research, relative lake volume (RLV) is calculated in two steps. The first step is to identify individual lakes, determine their analysis extents, and calculate annual lake area from Landsat imagery. The second step is to derive lake area-elevation relationship, estimate lake surface elevation, and calculate lake water volume change. Details in the first step are shown in Fig. 2, which include three sub-steps: lake identification, lake analysis extent and seed determination, water classification and segmentation, and annual lake area calculation. For the second step, it can be divided into the derivation of the lake area-elevation relationship and calculating lake RLV.

### Lake analysis extent and seed determination

**Fig. 3:**
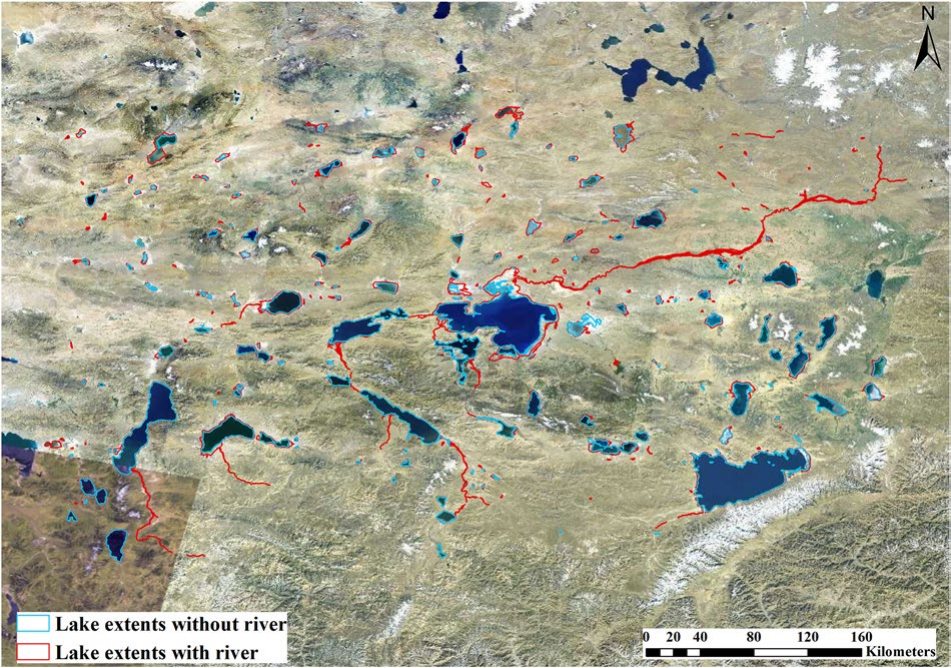
Lakes extents with and without river parts. Background remote sensing image is from http://t0.tianditu.gov.cn/img_c/wmts.

**Fig. 4:**
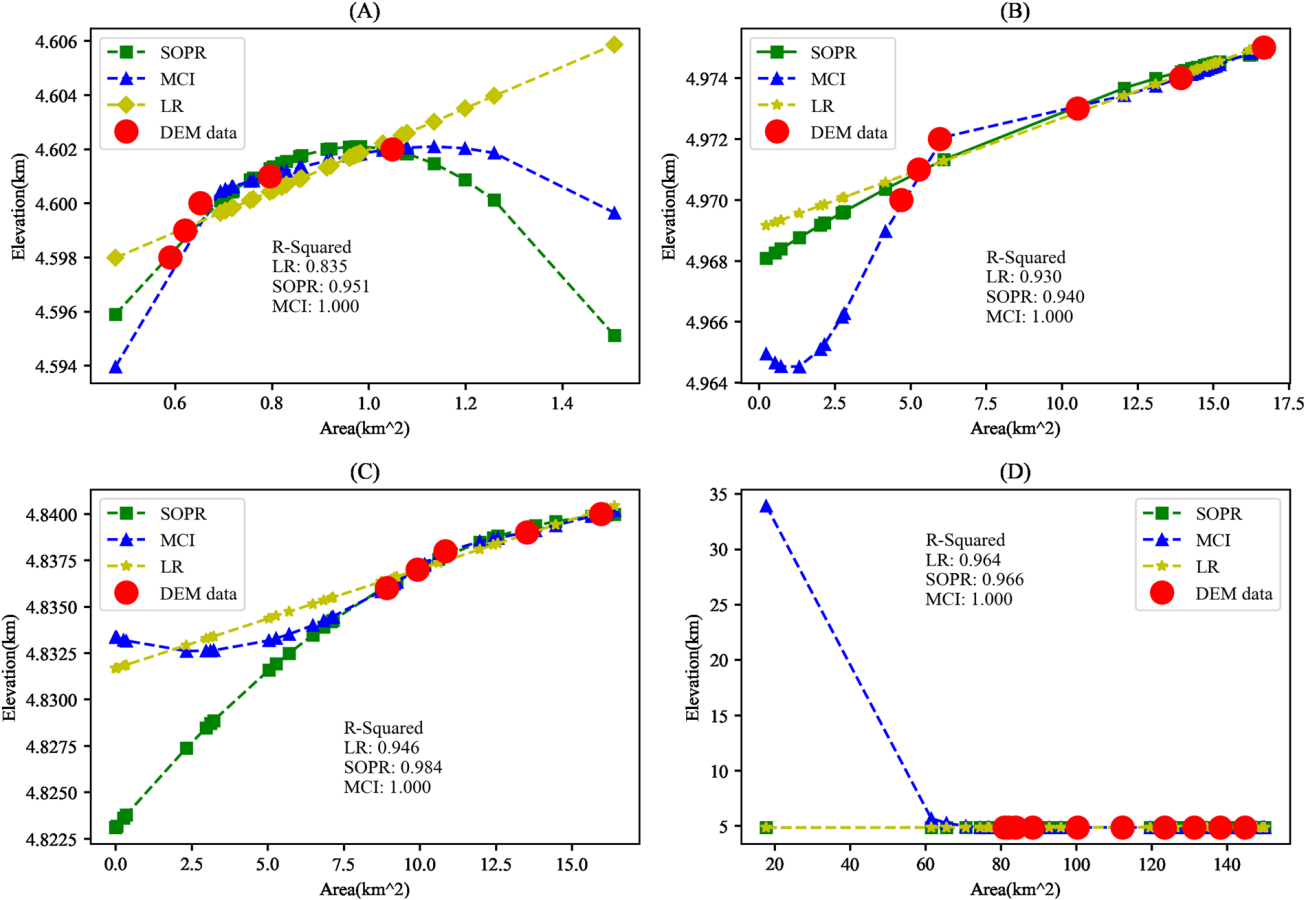
Four examples of estimating lake surface elevation based on the relationship between elevation and lake image area derived using the LR, SOPR and MCI data fitting methods. The elevation-lake area data pairs obtained from the SRTM DEM are shown as red points. The MCI method performs the best in (**A**) and (**C**) but may overfit in (**B**) and (**D**). See text for the performance of the LR and SOPR methods.

We used the JRC-GSW data^27^ to identify and select the lakes on the EBTP. Specifically, we used the max extent band to determine the maximum water extent between 1984 and 2020. From those maximum water extent pixels, spatially connected pixels were identified as individual waterbodies and those with an area larger than 1 km^2^ were kept. Those waterbodies include both lakes and the rivers connected with them, especially for large lakes (Fig. 3). The border between lakes and rivers is hard to define. The main body of a lake should have a slope close to zero when its slope is derived from SRTM DEM as the lake elevation is usually hydro-flattened in the DEM. We calculated the slope of each waterbody pixel using SRTM DEM and removed those pixels with a slope greater than 0°, which are considered as rivers. In this step, several patches of waterbody pixels may occur. We visually inspected those patches and only kept the patch that represents the main body of the lake associated with the waterbody as the approximate extent of the lake. This approach worked effectively for waterbodies larger than 50 km^2^ and 490 lakes and their extents were identified this way. In the process, we found there is one river linking two lakes from high resolution remote sensing images (see Fig. 4). The river and the lakes were kept as one lake in this study though the lakes were treated as separate lakes in former studies^1,19^. The above procedure, however, tends to remove many small waterbodies entirely. So for waterbodies less than 50 km^2^, we inspected each waterbody visually, identified 486 more lakes, and manually drew their lake approximate extents. Altogether, we identified a total of 976 lakes and their approximate extents on the EBTP. A 5-km buffer was generated for each lake and was used as the analysis extents for the lake to reduce computing resources and improve efficiency.

In addition to lake analysis extents, a point is also created for each lake (hereafter lake seed) to distinguish the target lake from other waterbodies within its analysis extent. The centroid point of each lake’s approximate extent was calculated as initial lake seed location but they were manually checked and edited, if necessary, to make sure they are inside their lake approximate extents.

### Water segmentation

Although the JRC-GSW data provide global monthly surface water map, it is not designed for mapping alpine lakes specifically. As such, we developed our own method for mapping lakes on the EBTP from Landsat imagery.

Since there are more than 30,000 Landsat images in this study area within the study period, Google Earth Engine (GEE)^31^ was used for image processing and data analysis. We first selected Landsat images between June and November in each year to exclude images with snow and ice. Landsat quality assessment band (hereafter BQA band) was used to remove cloud, shadow, saturation (for Landsat 5, 7 and 8) and terrain occlusion (for Landsat 8 only) pixels on each image. A composite image was then generated with these selected images for each year. In GEE, the composite images were generated using the SimpleComposite function. The function computes a Landsat top of atmosphere (TOA) composite from a collection of Landsat scenes. It calculates a cloud score (between 0 and 100) at each pixel for each image, selects the pixels with a cloud score less than a certain threshold, and calculates a percentile pixel value for the composite image. More details on the function can be found at https://developers.google.com/earth-engine/guides/landsat#simple-composite. In this research, we used a cloud score threshold of 10 and a percentile value of 0 (i.e. use the lowest pixel values with a cloud score of 10 or less). Because the water usually has lower pixel value than land, the composite images represent the maximum water extents in each year.

With the annual composite images, lake water pixels are classified using normalized difference water index (NDWI)^32^:

1 $${\rm{NDWI}}=\frac{{B}_{G}-{B}_{NIR}}{{B}_{G}+{B}_{NIR}}$$

where *B*_*G*_ and *B*_*NIR*_ are the green and near infrared bands, which are band 2 and 4 for Landsat 5/7 and bands 3 and 5 for Landsat 8 OLI images, respectively. Several other indexes have been used for lake mapping, such as modified NDWI (MNDWI)^33^, normalized difference moisture index (NDMI)^34^, and water ratio index (WRI)^34,35^. We chose NDWI in this study as existing research indicated that NDWI appears to be simple but more robust than the other index-based methods in detecting lake extent under various water conditions^36,37^.

Thresholding (or segmentation) is a key step in extracting water pixels from NDWI images. Usually, pixels with a NDWI value greater than 0 are considered as water. However, because of disparate geographical environment and dynamic water conditions, it is impossible to use the same NDWI threshold for all the lakes in all the years. In this research, we used local Otsu method^38,39^ to dynamically segment NDWI images. The Otsu method searches for the threshold that minimizes the intra-class variance of water pixels and non-water pixels, defined as a weighted sum of variances of the two classes:

2 $${\sigma }_{\omega }^{2}\left(t\right)={\omega }_{0}\left(t\right){\sigma }_{0}^{2}\left(t\right)+{\omega }_{1}(t){\sigma }_{1}^{2}(t)$$

where weights *ω*_0_ and *ω*_1_ are the probabilities of the two classes separated by a threshold t, and ${\sigma }_{0}^{2}$ and ${\sigma }_{1}^{2}$ are the variances of these two classes^38^. There are actually two options to find the threshold^38^. The first is to minimize the within-class variance defined in Eq. 2, and the second approach is to maximize the between-class variance using the equation below:

3 $${\sigma }_{b}^{2}\left(t\right)={\omega }_{1}(t){\omega }_{2}(t){\left[{\mu }_{1}\left(t\right)-{\mu }_{2}(t)\right]}^{2}$$

where *μ*_*i*_ is a mean of class i.

In this study, a Canny edge detection algorithm^40^ was first used to extract lake shorelines from NDWI images (see the yellow box in Fig. 2). A 120-m buffer was then generated around the shorelines. After that Otsu method was applied to the NDWI value within the buffer to obtain the optimal threshold that separates water from no-water pixels.

### Annual lake area

As water level changes, some lakes may have several separate waterbodies in some years due to reduced water volume. To handle this situation, we unioned all the annual water pixels within a lake’s analysis extent and, from which, we then identified the spatially connected water region which contains the lake’s seed as the lake’s maximum water extent during the study period for each lake. The maximum lake water extent is then used to identify annual lake water pixels and to calculate annual lake area (see red box in Fig. 2). In this way, even if a lake has separate waterbodies in some of the years, all the constituent waterbodies are counted as parts of the same lake.

We used the imagery from Landsat-5 TM (1984–2012), Landsat-7 ETM + (1999-), and Landsat-8^41^. When imagery from multiple sensors (Landsat 5 & 7 and 7 & 8) are available, lake area was calculated separately from each sensor and then combined. If the relative difference between the two sensors is within 2%, the average area is used for the year. Otherwise, annual Landsat composite images and lake boundaries were manually examined to decide which area is more accurate. In addition, annual lake area was manually checked if there is a significant change (>10%) from previous and following years. In rare cases, the composite images may have lakes frozen, covered by shadow (cloud or terrain) or dammed, which result in a large error in lake area. When this happened, we treated the composite image as contaminated and unreliable, and the lake area for the year was linearly interpolated using prior and later year’s lake area. Through those steps, we obtained the annual maximum lake area for each lake.

### Lake surface elevation

Lake surface elevation is essential to calculate water volume change. Both satellite altimetry and DTM have been used to estimate lake surface elevation^15,19,36^. While satellite altimetry is more accurate, it is limited to less than 170 large lakes on the TP^42–44^ and even fewer on the study area of this research^20^. Because of this, we used DTM data to estimate lake surface elevation.

Without lake bathymetry data, we can only estimate lake surface elevation based on the elevation-area relationship derived from DTM assuming that the slopes below and above the lake water surface when the DTM was collected are similar^11^. Some commonly used methods include linear equation^11^, second order parabolic equation^19^ and monotonic cubic spline fitting^1^. These methods have their own advantages and disadvantages. Because the linear regression is the simplest, it is usually suitable when fitting data is not enough for second order parabolic equation or mor complex methods. The second order parabolic equation is suitable for simulating the elevation-area relationship with small changes in slope. The monotonic cubic spline fitting, which constructs polynomial functions, can fit data more smoothly^45^ and model the elevation-area relationship with large slope changes^45^.

### Methods for deriving lake elevation-area relationship

Lake surface elevation can be estimated by calculating the average elevation of lake boundary^1,3,19,40^. This approach assumes that the DTMs are obtained before lake water volume started increase. The DTMs (SRTM and ALOS) we used were acquired in and after 2000^46,47^ and the surface area of some of the lakes is below the surface area when the DTMs were acquired. As such, lake surface elevation in this study is estimated based on the area-elevation relationship derived from the DTMs for each lake. Existing studies mainly used just one of a few methods, including linear equation^11^, parabolic equation^19^ or monotonic cubic spline fitting^1^, to derive the relationship. In this research, we used different methods under different situations.

Although existing research indicates that monotonic cubic interpolation (MCI) has the best performance in fitting elevation-area relationship^1^, we found that MCI may overfit. In this research, a combination of linear regression (LR), second order polynomial regression (SOPR), and MCI methods was used to derive the elevation-area relationship which was then used to estimate surface elevation with lake area obtained from Landsat imagery. The elevation-area pairs, where the elevation starts from the lowest elevation, increases at an interval of 1 m and stops at the highest elevation within a lake’s analysis extent, were obtained from SRTM and ALOS separately. At each elevation, pixels that are less than the elevation were extracted from DTM and converted into polygons. The maximum lake water extent is then used to select the polygons representing lake water surface areas and the sum of all the polygon area is the lake surface area for the elevation. The minimum (MinA) and maximum (MaxA) annual lake area from Landsat are then used to select the elevation-area pairs whose area is in the range of [MinA/1.5, MaxA*1.5] from both SRTM and ALOS, and the list with more elevation-area pairs is kept. If the two lists have the same length, the SRTM list is kept. The choice of the data fitting methods depends on the number of selected elevation-area pairs, which is discussed below and summarized in Table 3:

1. If the number of data pairs is zero or one, a new list of elevation-area pairs is generated from the selected DTM with three pairs whose area starts with MaxA*1.5. The LR method was then used to derive the elevation-area relationship. This approach is labelled as LRN;
2. If the number of data pairs is two, we used the LR to derive the elevation-area relationship and label this approach as LRC;
3. If the number of data pairs is equal to or greater than five and lake area range from the selected DTM fully covers the area range ([MinA, MaxA]) from Landsat imagery, the MCI method was used;
4. In other cases, the SOPR method was used. When the symmetric axis of the SOPR model falls within [MinA, MaxA], the derived elevation-area relationship will be non-monotonic. To avoid this, when the symmetric axis fell within [MinA, MaxA], the LR method was used instead and this is labeled as LRS.

**Table 3:** Selection of data fitting methods for deriving elevation-area relationship for each lake.

Conditions	Method	Abbreviation
The number of data pairs is 0 or 1	Generate 3 new data pairs and then use LR	LRN
Number of data pairs = two	LR	LRC
Number of data pairs >=≥ five and MinA is larger than the minimum area from DTM	MCI	MCI
None of the above	SOPR but use LR when the symmetric axis of SOPR falls within [MinA, MaxA]	SOPR/LRS

Four lakes, with the area ranging from 0.97 km^2^ to 149.3 km^2^, were selected to explain the typical situations when different methods were used. Figure 4 shows the elevation-area pairs (red points) from the DTMs and the elevations estimated based on Landsat image lake area using different data fitting methods. MCI has the best performance for the lakes in Fig. 4A,B and there is no obvious difference between SOPR and MCI in Fig. 4C,D. The LR has the worst performance in Fig. 4A–C. However, when the elevation-area pairs from the DTMs do not cover the lake area range from Landsat images, estimated elevation can have serious error, especially for MCI. Take the lake in Fig. 4B as an example, its area range from Landsat imagery is [0.23, 16.71] km^2^ from 1989 to 2019, yet the smallest area obtained from SRTM is 4.69 km^2^. This is because the SRTM was collected in 2000 but most lakes had been expending since 1995 in the region. While MCI fits generally well with the elevation-area pairs from the DTMs, elevations outside the DTM area range are estimated unreal in Fig. 4B,D, especially in Fig. 4D where the elevation of the lake area smaller than the smallest DTM area are estimated unreasonably high. Those examples indicate that MCI may overfit and should only be used when image lake area is within the DTM area range. SOPR also has the same issue when image lake area is outside DTM area range. As such, SOPR is only used when the minimum image lake area is smaller than the minimum DTM area. When using SOPR method, the fitted curve is not monotonic when its symmetric axis falls into [MinA, MaxA] (Fig. 4A). When this happens, LR method was used instead. In addition to the above situations, the number of elevation-area pairs from the DTMs which are within the image area range of [MinA/1.5, MaxA*1.5] also play a role as discussed in RLV calculation.

### Lake water volume

Relative lake volume (RLV) in year y is the lake water volume change between the year and the base year, which is 1989 in this study, and can be calculated by the integral of an elevation-area function:

4 $${{\rm{RLV}}}_{y}={\int }_{{E}_{1989}}^{{E}_{y}}AdE={\int }_{{E}_{1989}}^{{E}_{y}}f\left({\rm{E}}\right)dE$$

5 $$f\left({\rm{E}}\right)=A={\rm{a}}+{\rm{bE}}+{{\rm{cE}}}^{2}\;or\;d+eE$$

where E denotes lake surface elevation, and A is the lake area at the elevation. f(E) is the fitted elevation-area function using the LR or SOPR methods, and a, b, c, d, and e are the coefficients of the SOPR and LR models.

**Fig. 5:**
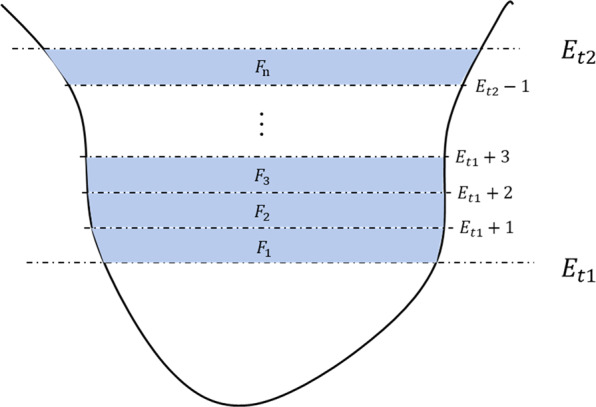
Schematic diagram showing how relative lake water volume can be calculated using a series of frustums. The volume between time t1 to t2 can be divided into a series of frustums (F_1_ to F_n_) with a height of 1 m. For each frustum, its volume can be calculated with its top and bottom area using Eq. 6.

Since the MCI function is not integrable analytically, we cut the lake water volume between two dates into frustums with 1 m of elevation intervals (Fig. 5). With an elevation list [${E}_{t1},{E}_{t1+1},{E}_{t1+2},\ldots ,{E}_{t2-1},{E}_{t2}$], the corresponding lake area was obtained using the fitted MCI. The RLV is the sum of all the frustums (i.e., ${\sum }_{1}^{n}{V}_{Fn}$), which can be calculated by the following equation:

6 $${V}_{F}=({A}_{U}+{A}_{D}+\sqrt{\left({A}_{u}+{A}_{D}\right)}\times \frac{1}{3}h)$$

where *A*_*U*_ and *A*_*D*_ denote the base and top surface area of a frustum and *h* denotes the height of the frustum, which is 1 m here. In this study, All RLVs were calculated relative to their lake water volumes in 1989. In another word, the RLVs of all the lakes are 0 in 1989.

## Data Records

A total of 976 annual lakes’ area and RLV data are provided for the entire EBTP for the period of 1989–2019. The dataset is available at the Zenodo repository as ESRI’s shapefiles and a summary table (10.5281/zenodo.7042325)^48^. The shapefiles are in the geographic coordinate system (ESPG: 4326). There are three shapefiles: relative lake volume, lake area, and lake seeds. *Relative_Lake_Volume.shp* is the shapefile containing the relative lake volume (RLV) of 976 lakes from 1989 to 2019 on the EBTP. The data fitting methods and the DEM data sources are also included as attributes. *Lake_Area.shp* is the shapefile which contains annual lake area of the 976 lakes from 1989 to 2019. Lake_Seeds.shp is the point shapefile containing the longitude and latitude of the lake seeds. Besides, the *Equation_Index.xlsx* has the elevation-area relationship equations used for each lake.

## Technical Validation

We compared the results in this study with a widely used lake surface elevation and storage dataset from the LEGOS Hydroweb^30^ as well as two most recent lake water volume data on the TP from Li, *et al*.^19^ (referred to as Li’s data hereafter) and Yao, *et al*.^1^ (referred to as Yao’s data hereafter). Because the volume data in this study are relative volume change to 1989, we recalculated both Li’s and Yao’s data to make sure their volume data are also relative to 1989. Pearson’s correlation coefficient (PCC) and symmetric mean absolute percentage error (sMAPE), which is defined in Eq. 7, were used to evaluate the accuracy.

7 $${\rm{sMAPE}}=\frac{1}{n}{\sum }_{i=1}^{n}\frac{2* | {x}_{i}-{y}_{i}| }{| {x}_{i}| +| {y}_{i}| }$$

where n is the sample size. *x*_*i*_ and *y*_*i*_ are the *i*th data value from this study and existing datasets, respectively. The range of sMAPE is [0, 2] and the smaller the sMAPE, the smaller the relative error. sMAPE is a scale-independent accuracy index based on percentage errors^49^. Compared with commonly used Root Mean Square Error (RMSE), sMAPE can be used to compare lakes with different magnitude of RLV. In addition, sMAPE allows 0 in the data, which is very common in RLV. In contrast, mean relative error (MRE) has issues when data values are 0. Because those reasons we used sMAPE instead of MRE here.

The Supplementary Table 1 shows the PCC and sMAPE when comparing our RLVs with Hydroweb (21 lakes) and Li’s data (40 lakes) for the overlapping lakes. All the PCCs are significant with p-values less than 0.01. Compared with Hydroweb data, 13 lakes (66.7%) have a PCC larger than 0.8 and a sMAPE less than 1. Compared with Li’s data, 31 lakes (77.5%) have a PCC larger than 0.8 and a sMAPE less than 1. Those results suggest that our RLVs match generally well with both Hydroweb and Li’s lake data.

**Fig. 6:**
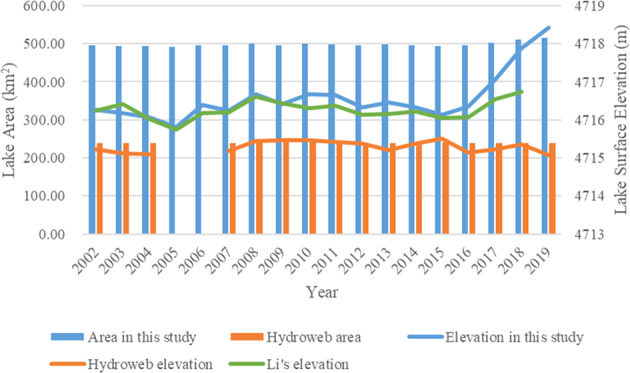
Comparison of lake area and lake surface elevation between the results in this study and two existing data (Hydroweb and Li’s data) for lake Ngangla-Ringco from 2002 to 2019. The y-axis on the left, representing lake area, is for the vertical bars. The y-axis on the right, representing lake surface elevation, is for the horizontal lines.

There are discrepancies among the datasets. For example, lake Ngangla-Ringco has a PCC of −0.140 and sMAPE of 1.263 when compared with Hydroweb data but a PCC of 0.811 and sMAPE of 1.140 when compared with Li’s data. Three lakes (Ngangla-Ringco, Gozha-Co and Taro-Co), which have either the least PCC or the largest sMAPE with Hydroweb or Li’ data (bold and italic in Supplementary Table 1), were further examined. For Ngangla-Ringco, Fig. 6 shows the differences in lake area and surface elevation between our data and the two existing datasets. From 2016 to 2019, while our and Li’s lake surface elevation both show a significant increase, Hydroweb elevation has a slight decrease. From 2002 to 2019, our lake area is around 500 km^2^ but Hydroweb lake area is about 240 km^2^, only about the half of our lake surface area.

**Fig. 7:**
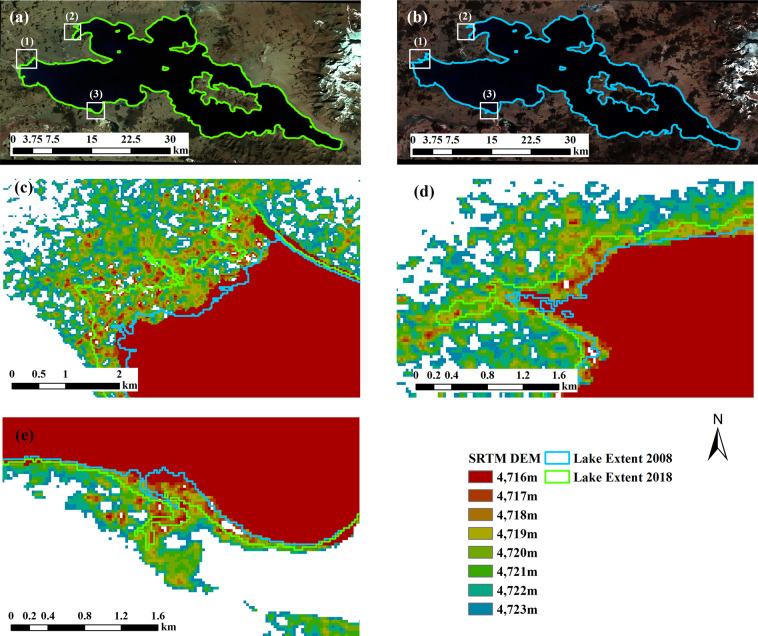
Lake extents in 2018 (**a**) and 2008 (**b**) and in three close-up areas (**c**), (**d**) and (**e**) (corresponding to boxes (1), (2), (3) in (**a**) and (**b**), respectively) from the results in this study for lake Ngangla-Ringco. Images in (**a**) and (**b**) are composite image (R: Near-infrared band, G: Red band, B: Green band) from Landsat 5 and Landsat 8 respectively. DEM shown in (c)-(e) are SRTM DEM.

The boundaries of lake Ngangla-Ringco in 2008 (before significant increase in 2016) and 2018 (after significant increase in 2016) are shown in Fig. 7 with SRTM DEM added to help examine lake boundary elevation. The mean lake boundary elevations are 4716.68 and 4717.88 meters in 2008 and 2018 respectively and Fig. 7c–e show a clear increase in surface elevation (as shown by the lake shoreline location) between the years. In addition, our lake boundaries (Fig. 7a and b) fit well visually with the lake on the composite images, indicating our lake areas are more reliable than Hydroweb data for the lake.

**Fig. 8:**
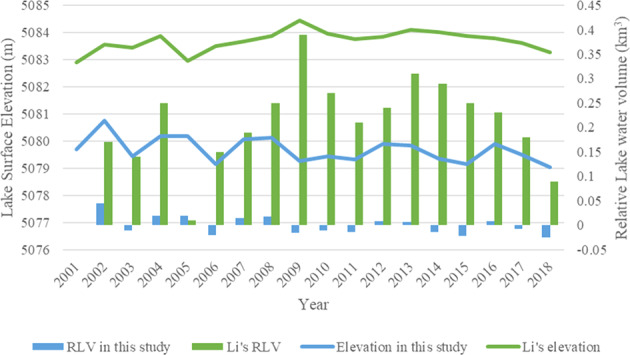
Comparison of relative lake water volume and lake surface elevation between the results in this study and two existing data (Hydroweb and Li’s data) for lake Gozha-Co from 2001 to 2018. The y-axis on the left, representing lake surface elevation, is for the lines. The y-axis on the right, representing relative lake water volume, is for the vertical bars.

In Li’s data, lake Gozha-Co’s surface elevation rose from 2001 to 2009 reaching the highest elevation of 5084.43 m in 2009, and then it started a decreasing trend (Fig. 8). In our data, lake surface elevation fluctuated but generally had been decreasing from 2001 to 2018. While our lake surface elevation ranges between 5079 and 5081 m, Li’s lake elevation is between 5083 m and 5085 m, which leads to extremely larger lake water volume compared with our data.

**Fig. 9:**
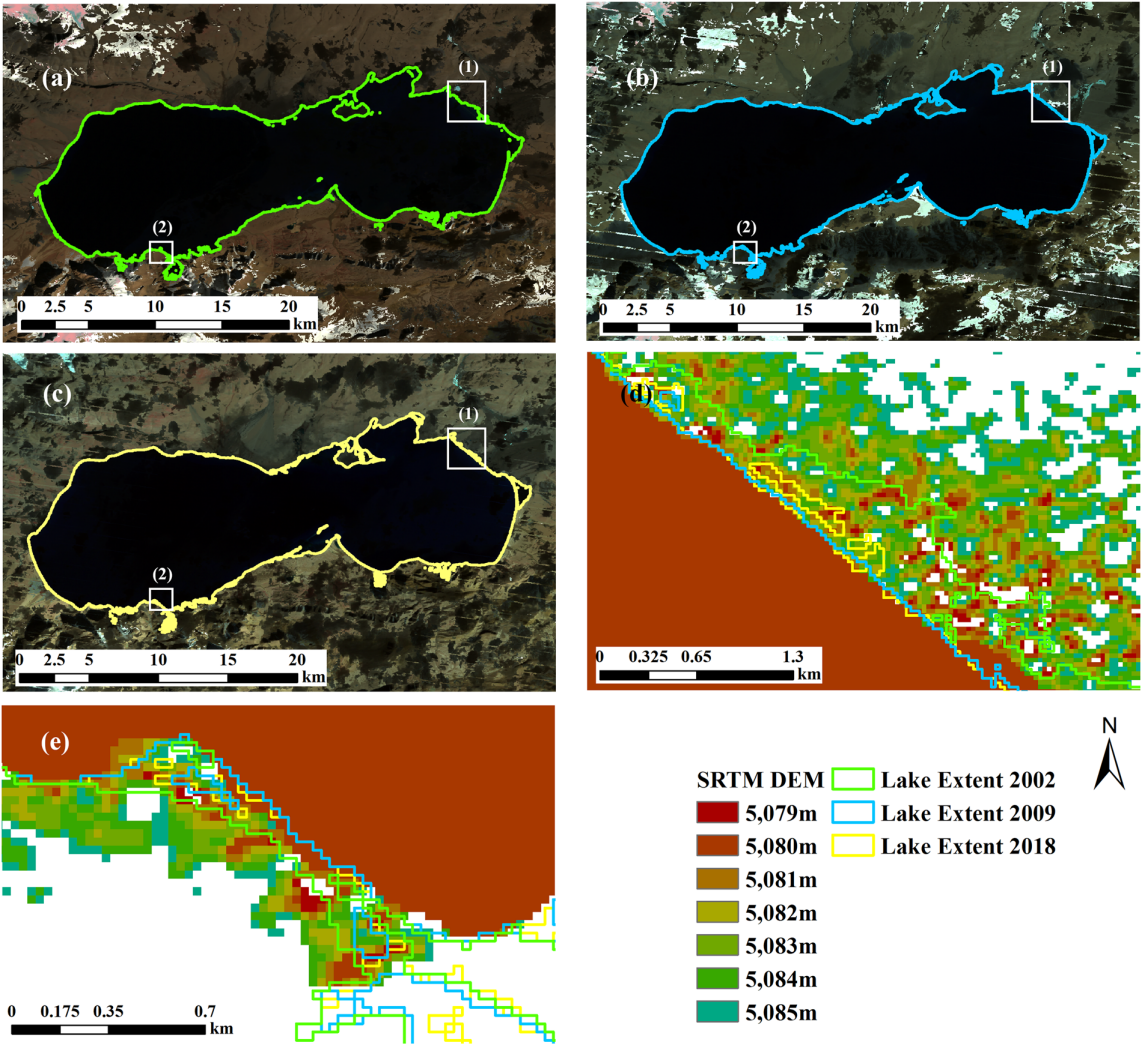
Lake extents in 2002 (**a**), 2009 (**b**), and 2018 (**c**) and two close-up areas (**d**), and (**e**) (corresponding to boxes (1) and (2) in image (**a**), (**b**) and (**c**), respectively) from the results in this study for lake Gozha-Co. DEM shown in (**d**) and (**e**) are SRTM DEM. Composite images in (**a**–**c**) (R: Near-infrared band, G: Red band, B:Green band) are from Landsat 7.

For further assessment, lake Gozha-Co’s extents (Fig. 9a–c) in 2002, 2009, and 2018 and SRTM DEM are shown in Fig. 9. The mean lake boundary elevations are 5080.74 m, 5079.28 m and 5079.04 m in 2002, 2009 and 2018 respectively and Fig. 9d,e show no significant change in surface elevation in those years and the highest elevation occurred in 2002 rather than in 2009 and the elevation in 2009 and 2018 does not differ much. All those confirm that our data are more reliable than Li’s data. The large difference between our and Li’s volume data is probably caused by the difference in elevation. Unfortunately, Li’s data doesn’t provide lake area.

**Fig. 10:**
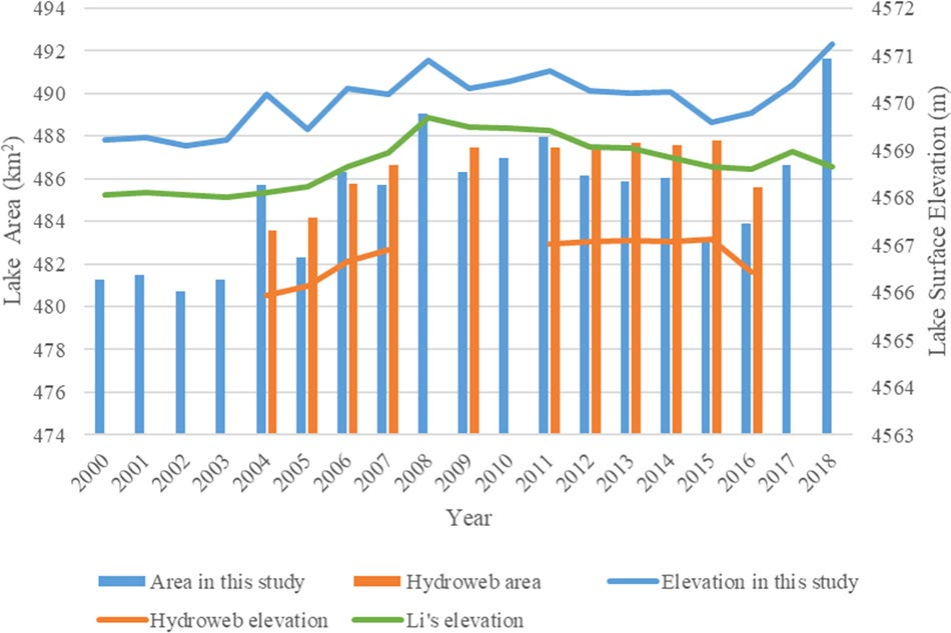
Comparison of lake area and lake surface elevation between the results in this study and two existing data (Hydroweb and Li’s data) for lake Taro-Co from 2000 to 2018. The y-axis on the left, representing lake area, is for the vertical bars. The y-axis on the right, representing lake surface elevation, is for the lines.

Figure 10 shows the differences in lake area and surface elevation among the datasets for lake Taro-Co. Our data and the two existing datasets generally have a similar increase trend in surface elevation in 2004–2008. Our surface elevation have been increasing from 2015 to 2018 but Hydroweb and Li’s elevation experienced a decrease from 2015 to 2016 and Li’s elevation also decreased from 2017 to 2018. In addition, both our area and elevation data fluctuated more than the other two datasets.

**Fig. 11:**
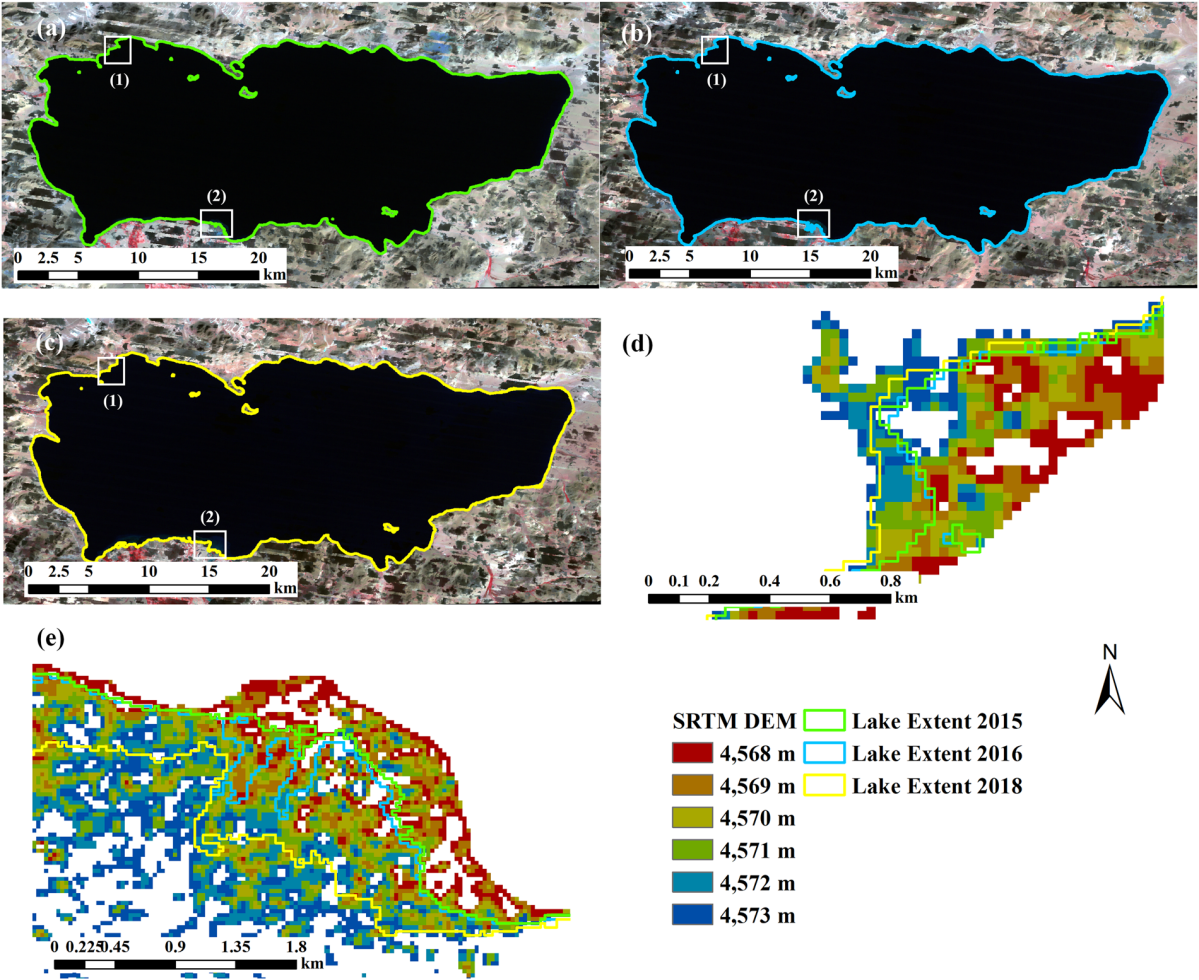
Lake extents from this study for lake Taro-Co in 2015 (**a**), 2016 (**b**), and 2018 (**c**) and two close-up areas (**d**), and (**e**) (corresponding to boxes (1) and (2) in image (**a**,**b**) and (**c**), respectively). DEM shown in (**d**) and (**e**) are SRTM DEM. Composite images in (**a**–**c**) (R: Near-infrared band, G: Red band, B:Green band) are from Landsat 7.

The extents (Fig. 11a–c) for lake Taro-Co in 2015, 2016, and 2018 and SRTM DEM are shown in Fig. 11. The mean elevation of the lake boundaries from this study are 4569.59 m, 4569.77 m, and 4571.25 m, respectively. A significant increase in lake surface elevation in 2018 can be clearly observed in Fig. 11d,e.

**Fig. 12:**
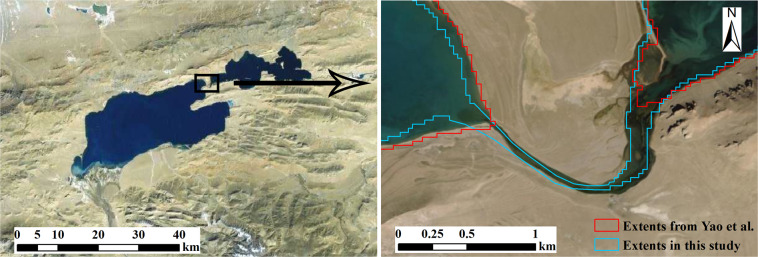
An example that connected waterbodies were counted as one lake (lake ID of L016) in our study but separate lakes (Qiagui Co (west) and Wuru Co (east)) in Yao’s data. Remote sensing image is from http://t0.tianditu.gov.cn/img_c/wmts. Background remote sensing image is from http://t0.tianditu.gov.cn/img_c/wmts.

Yao, *et al*.^1^ also published a lake storage data on the IB. Their datasets include the annual RLV for 871 lakes with an area larger than 1 km^2^ from 2009 to 2015, and the annual RLV for 126 lakes with an area larger than 50 km^2^ from 2002 to 2015. Among these lakes, we found 816 overlapping lakes compared with their lakes larger than 1 km^2^, and all lakes larger than 50 km^2^. Our data have less lakes than Yao’s data on the IB primarily because that connected waterbodies were counted as separate lakes in Yao’s data as shown in the example in Fig. 12.

For lakes larger than 1 km^2^, 447 out of 816 (54.78%) lakes have PCCs with p-value less than 0.05. The average PCC and sMAPE for these 447 lakes are 0.882 and 0.722, respectively. In addition, 301 lakes have PCC ≥ 0.8 and sMAPE < 1. For lakes larger than 50 km^2^, 110 out of 126 (87.30%) lakes have PCCs with p-value less than 0.05. The average PCC and sMAPE for these 110 lakes are 0.883 and 0.609, respectively. In addition, 73 lakes have PCC ≥ 0.8 and sMAPE < 1. Overall, the data in this study match better with Yao’s 50km^2^ data than 1 km^2^ data. Because Yao *et al*.^1^ did not provide lake area and surface elevation data, it is difficult for us to further examine the discrepancy.

## Usage Notes

**Fig. 13:**
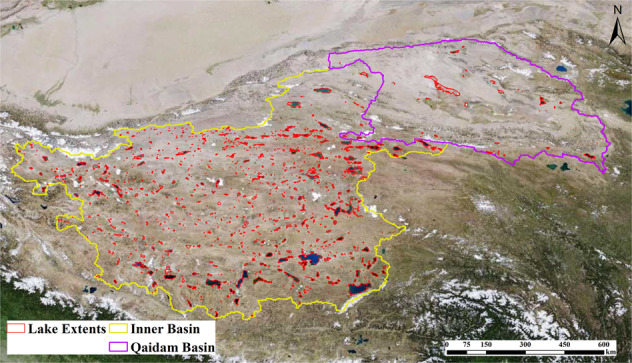
A total of 976 lakes larger than 1 km^2^ identified on the EBTP. Remote sensing image is from http://t0.tianditu.gov.cn/img_c/wmts.

We identified a total of 976 lakes on the EBTP, and their maximum extents during the study period are shown in Fig. 13. 930 of those lakes (95.29%) are located on the Inner Basin, and only 46 (4.71%) are on the Qaidam Basin. Large lakes are primarily located on the southern and eastern periphery of the inner basin.

Hydrologically open and closed lakes may change differently. While individual lake changes may present conflicting signals (i.e., open lakes have limited changes and downstream closed lakes have large changes), combining hydrologically connected lakes as one lake may alleviate this issue when studying the changes in lake water storage^50^.

In this study, we combined the lakes that are connected by short rivers as one lake (see Fig. 12 for an example). Most of our connected lakes, however, are “hydrologically connected” through low terrain. These lakes are formed when small separate waterbodies of a large lake in dry periods become connected with inc km^2^ease in water level during wet periods (e.g., several small lakes near the Lake Selin Co). In this case, it is impractical to separate these lakes during wet periods.

Our water volume dataset is aimed for studying the lake water changes in the hydrological system instead of individual lakes. From the water balance perspective, such studies are best done using hydrologically connected lakes, ideally closed basins, as study units. Combining hydrologically connected lakes in the same basin could help us understand lake water storage condition at basin scale and how lake water storage responds to external environmental forcing (e.g., variation in precipitation and glacier)^51^.

This research provides a census on water volume change for the lakes greater than or equal to 1 km^2^ on the EBTP from 1989–2019 using Landsat imagery and DTM data. The dataset in this study, compared with other existing data, covers more lakes, especially small lakes in 1–10 km^2^, and longer time period. Comparison with three major existing data products in the region indicates that our dataset is reliable and might be more accurate. To the best of our knowledge, the dataset in this study provides the longest and most comprehensive lake water volume change data in the region, especially for small lakes (1–10 km^2^). The dataset is valuable in studying the impacts of climate change and water balance in the region. The workflow used in this study can be further improved to process individual Landsat image (instead of annual composite image) and create a lake water volume dataset with a higher temporal resolution in the future.

## Supplementary information

Supplementary Table 1

## Data Availability

The annual area and RLV of lakes larger than 1 km^2^ from 1989 to 2019 were produced using GEE API and Python. The code developed for this work are openly shared with the scientific community at Zenodo repository^48^. GEE should be used to access and edit the code.

## References

[1] Yao F (2018). Lake storage variation on the endorheic Tibetan Plateau and its attribution to climate change since the new millennium. Environ. Res. Lett..

[2] Williamson CE, Saros JE, Vincent WF, Smol JP (2009). Lakes and reservoirs as sentinels, integrators, and regulators of climate change. Limnol. Oceanogr..

[3] Yang K (2017). Recent dynamics of alpine lakes on the endorheic Changtang Plateau from multi-mission satellite data. J. Hydrol.: X.

[4] Qiu J (2008). China: the third pole. Nature.

[5] Field, C. B. *Climate Change 2014–Impacts, Adaptation and Vulnerability: Regional Aspects*. (Cambridge University Press, 2014).

[6] Hansen J, Ruedy R, Sato M, Lo K (2010). Global surface temperature change. Rev. Geophys..

[7] Xu Z, Gong T, Li J (2008). Decadal trend of climate in the Tibetan Plateau—regional temperature and precipitation. Hydrol. Process..

[8] Lei Y (2017). Lake seasonality across the Tibetan Plateau and their varying relationship with regional mass changes and local hydrology. Geophys. Res. Lett..

[9] Liu J, Wang S, Yu S, Yang D, Zhang L (2009). Climate warming and growth of high-elevation inland lakes on the Tibetan Plateau. Glob. Planet. Change.

[10] Boos WR, Kuang Z (2010). Dominant control of the South Asian monsoon by orographic insulation versus plateau heating. Nature.

[11] Yang R (2017). Spatiotemporal variations in volume of closed lakes on the Tibetan Plateau and their climatic responses from 1976 to 2013. Clim. Change.

[12] Ma R (2010). A half‐century of changes in China’s lakes: Global warming or human influence?. Geophys. Res. Lett..

[13] Zhang G (2017). Extensive and drastically different alpine lake changes on Asia’s high plateaus during the past four decades. Geophys. Res. Lett..

[14] Donchyts G (2016). Earth’s surface water change over the past 30 years. Nat. Clim. Chang..

[15] Song C, Huang B, Ke L (2014). Inter‐annual changes of alpine inland lake water storage on the Tibetan Plateau: Detection and analysis by integrating satellite altimetry and optical imagery. Hydrol. Process..

[16] Song C, Sheng Y, Ke L, Nie Y, Wang J (2016). Glacial lake evolution in the southeastern Tibetan Plateau and the cause of rapid expansion of proglacial lakes linked to glacial-hydrogeomorphic processes. J. Hydrol.: X.

[17] Song C (2017). Heterogeneous glacial lake changes and links of lake expansions to the rapid thinning of adjacent glacier termini in the Himalayas. Geomorphology.

[18] Wan W (2016). A lake data set for the Tibetan Plateau from the 1960s, 2005, and 2014. Sci. Data.

[19] Li X (2019). High-temporal-resolution water level and storage change data sets for lakes on the Tibetan Plateau during 2000–2017 using multiple altimetric missions and Landsat-derived lake shoreline positions. Earth System Science Data.

[20] Zhang G (2017). Lake volume and groundwater storage variations in Tibetan Plateau’s endorheic basin. Geophys. Res. Lett..

[21] Zhou J (2015). Exploring the water storage changes in the largest lake (Selin Co) over the T ibetan P lateau during 2003–2012 from a basin‐wide hydrological modeling. Water Resour. Res..

[22] Zhang Y, Zhang G, Zhu T (2020). Seasonal cycles of lakes on the Tibetan Plateau detected by Sentinel-1 SAR data. Sci. Total Environ..

[23] Huang H (2017). Mapping major land cover dynamics in Beijing using all Landsat images in Google Earth Engine. Remote Sens. Environ..

[24] Google Earth Engine. USGS Landsat 5 TM Collection 1 Tier 1 Raw Scenes. https://explorer.earthengine.google.com/#detail/LANDSAT%2FLT05%2FC01%2FT1 (2020).

[25] Google Earth Engine. USGS Landsat 7 Collection 1 Tier 1 and Real-Time data Raw Scenes. https://explorer.earthengine.google.com/#detail/LANDSAT%2FLE07%2FC01%2FT1_RT (2020).

[26] Google Earth Engine. USGS Landsat 8 Collection 1 Tier 1 and Real-Time data Raw Scenes. https://explorer.earthengine.google.com/#detail/LANDSAT%2FLC08%2FC01%2FT1_RT (2020).

[27] Pekel J-F, Cottam A, Gorelick N, Belward AS (2016). High-resolution mapping of global surface water and its long-term changes. Nature.

[28] Farr TG (2007). The shuttle radar topography mission. Rev. Geophys..

[29] Tadono T (2014). Precise global DEM generation by ALOS PRISM. ISPRS Annals of the Photogrammetry, Remote Sensing and Spatial Information Sciences.

[30] Crétaux J-F (2011). SOLS: A lake database to monitor in the Near Real Time water level and storage variations from remote sensing data. Adv. Space Res..

[31] Gorelick N (2017). Google Earth Engine: Planetary-scale geospatial analysis for everyone. Remote Sens. Environ..

[32] Gao B-C (1996). NDWI—A normalized difference water index for remote sensing of vegetation liquid water from space. Remote Sens. Environ..

[33] Weekley D, Li X (2019). Tracking multidecadal lake water dynamics with Landsat imagery and topography/bathymetry. Water Resour. Res..

[34] Elsahabi M, Negm A (2016). & El Tahan, A. H. M. Performances evaluation of surface water areas extraction techniques using Landsat ETM+ data: case study Aswan High Dam Lake (AHDL). Procedia Technology.

[35] Barbieux K, Charitsi A, Merminod B (2018). Icy lakes extraction and water-ice classification using Landsat 8 OLI multispectral data. Int. J. Remote Sens..

[36] Qiao B, Zhu L, Yang R (2019). Temporal-spatial differences in lake water storage changes and their links to climate change throughout the Tibetan Plateau. Remote Sens. Environ..

[37] Rokni K, Ahmad A, Selamat A, Hazini S (2014). Water feature extraction and change detection using multitemporal Landsat imagery. Remote sensing.

[38] Otsu N (1979). A threshold selection method from gray-level histograms. IEEE Trans. Syst. Man Cybern..

[39] Setiawan, B. D., Rusydi, A. N. & Pradityo, K. Lake edge detection using Canny algorithm and Otsu thresholding. *2017 International Symposium on Geoinformatics (ISyG)*. 72–76 (2017).

[40] Bao P, Zhang L, Wu X (2005). Canny edge detection enhancement by scale multiplication. IEEE Trans. Pattern Anal. Mach. Intell..

[41] Cristóbal J, Jiménez‐Muñoz J, Sobrino J, Ninyerola M, Pons X (2009). Improvements in land surface temperature retrieval from the Landsat series thermal band using water vapor and air temperature. J. Geophys. Res.-Atmos..

[42] Hwang C (2019). Lake level changes in the Tibetan Plateau from Cryosat-2, SARAL, ICESat, and Jason-2 altimeters. Terr. Atmos. Ocean Sci.

[43] Jiang L, Nielsen K, Andersen OB, Bauer-Gottwein P (2017). Monitoring recent lake level variations on the Tibetan Plateau using CryoSat-2 SARIn mode data. J. Hydrol.: X.

[44] Li H, Qiao G, Wu Y, Cao Y, Mi H (2017). Water level monitoring on Tibetan lakes based on ICESat and ENVISAT data series. International Archives of the Photogrammetry, Remote Sensing & Spatial Information Sciences.

[45] Gray DK, Hampton SE, O’Reilly CM, Sharma S, Cohen RS (2018). How do data collection and processing methods impact the accuracy of long‐term trend estimation in lake surface‐water temperatures?. Limnol. Oceanogr. Meth..

[46] Takaku, J., Tadono, T. & Tsutsui, K. Generation of high resolution global DSM from Alos PRISM. *The International Archives of the Photogrammetry, Remote Sensing and Spatial Information Sciences* **XL-4** (2014).

[47] Van Zyl JJ (2001). The Shuttle Radar Topography Mission (SRTM): a breakthrough in remote sensing of topography. Acta Astronaut..

[48] Wang J (2022). Zenodo.

[49] Chen C, Twycross J, Garibaldi JM (2017). A new accuracy measure based on bounded relative error for time series forecasting. PloS one.

[50] Dong S, Peng F, You Q, Guo J, Xue X (2018). Lake dynamics and its relationship to climate change on the Tibetan Plateau over the last four decades. Reg. Envir. Chang..

[51] Yan D (2019). A data set of inland lake catchment boundaries for the Qiangtang Plateau. Sci. Data.

[52] Ke L (2022). Constraining the contribution of glacier mass balance to the Tibetan lake growth in the early 21st century. Remote Sens. Environ..

[53] Luo S (2022). Satellite laser altimetry reveals a net water mass gain in global lakes with spatial heterogeneity in the early 21st century. Geophys. Res. Lett..

